# Environmental Risks Associated with Symptoms of Acute Respiratory Infection among Preschool Children in North-Western and South-Southern Nigeria Communities

**DOI:** 10.3390/ijerph14111396

**Published:** 2017-11-16

**Authors:** Oluwafunmilade A. Adesanya, Chi Chiao

**Affiliations:** 1 Ogun State Government and UPLIFT Development Foundation, Abeokuta 110001, Nigeria; dejilade@yahoo.com; 2 Institute of Health and Welfare Policy, College of Medicine, National Yang-Ming University, No. 155, Sec. 2, Li-Nong St., Taipei 112, Taiwan

**Keywords:** regional disparities, acute respiratory infections, environmental risks, children, Nigeria

## Abstract

The exposure-disease-stress model places young children in their physical and social contexts and considers the extent and intensity of associational links to symptoms of acute respiratory infection (ARI), taking in to account a range of biological, social, and environment components. This study uses the 2013 Nigeria Demographic and Health Survey to assess the individual and environmental risks present in the North-Western and South-Southern Nigerian communities and examines their associations with ARI symptoms. The descriptive findings show that the prevalence of ARI symptoms is significantly higher among preschool children in the North-Western province (5.7%) than in the South-Southern province (1.4%) (*p* < 0.001). In addition to regional differences, multilevel logistic models further indicate that the increased likelihood of a child suffering from ARI symptoms is significantly associated with the dry season (aOR 1.42; 95% CI: 1.02–1.97) and household poverty (aOR 1.42; 95% CI: 1.01–1.99), even after adjusting for the cooking fuel used and various other characteristics of the children, households, and communities. These findings underscore the importance of taking into account environmental risks when addressing specific regional variations in ARI symptoms, because these determinants differ between communities in Nigeria. As it is imperative to achieve minimum levels of child health, in order to improve economic development across regions, future health policies aiming to promote child health will benefit from taking a region-specific perspective into consideration.

## 1. Background

Over many years, efforts have been made by international communities with the aim of improving child health; however, environmental health risk, which is known to be associated with the health of preschool age children and occurs predominately in low and middle income countries (LMICs), still remains a serious concern [1,2]. This is because it is limited to children living in certain vulnerable environmental conditions. It is specifically a problem for populations living in disadvantaged communities, as these communities face a greater likelihood of exposure to environmental hazards, due to rises in desertification, urbanization, deforestation, overpopulation and pollution—which have become important paradigms in Nigeria in the last three decades [3,4,5]. Sub-Saharan Africa (SSA) is the highest regional contributor to under-five mortality and acute respiratory infection (ARI), with over a million deaths of young children in 2015 [6]. 

UNICEF reported that nine out of 10 children living in extreme poverty were from SSA, as this region is also the home of the majority of the world’s pre-school age children. Compared to children from richer households, children from poor households are more likely to be exposed to poor environmental conditions, such as indoor air pollution due to cooking fuels, that may exacerbate childhood diseases, specifically ARI symptoms [6,7].

Prior research on ARI has often centered on the household characteristics of the children being studied [8]. A systematic review by Gee and his colleagues, that included fifteen systematic reviews, six meta analyses, and fourteen intervention studies, identified consistent evidence that exposures to environmental air pollutants, second hand smoke, inhaled chemicals, and respiratory viruses increased the risk of respiratory infection [4]. A case-control study linked exposure to environmental risks—namely overcrowding, keeping pets in the home, use of biomass fuel and use of a lantern at night—to ARI hospitalized cases [9]. Going beyond ARI symptoms that involve a correlation with mother-related and household-related factors, a handful of studies have suggested that community factors also have potential role in whether a child exhibits ARI symptoms [10]. Considering also the childhood characteristics, the status of orphan and vulnerable children (OVC) had an important role in the relationships between the proximate/socio-economic determinants and poor health outcomes in SSA. OVC status places the child in peril and being OVC puts a child at greater risk of ARI, due to their limited access to medical care, such as vaccinations. Recent research on child health has further incorporated the social environment and demonstrated that the community OVC rate has an overarching influence on shaping a child’s health [11]. 

In line with empirical evidence, we hypothesized that seasonal variation, as well as region specific individual and environmental factors, interplays synergistically to produce various different regional stressors that act to exacerbate differential effects on children at risk of developing ARI symptoms [4]. Specific attention needs to be paid to the *environmental conditions* that are related to the child’s geographical residence. In SSA, regional factors, coupled with increased economic expansion, an acceleration in urbanization, the presence of environmental hazards, and various biological mechanisms, are likely to lead to children experiencing differential exposure to health risks—such is the case with Nigeria, where its North-Western and South-Southern geo-political provinces have different levels of dust exposure [12]. Furthermore, the South-Southern community is exposed to a higher level of water pollution due to oil spillage, which is coupled with air pollution due to gas flaring [13]. 

The present study builds on the exposure-disease-stress model [4], to explore the variation in ARI symptoms and does this by examining the province-specific individual and environmental risk factors. The theoretical basis of this study emphasizes the idea that the differential experiences of individuals are based on societal factors related to each individual province and that these shape the household characteristics within these communities. Households within the catchment area of a given province may provide counterbalancing resources that can modify household characteristics and act to shape the exposed children’s health [4]. We proposed that the extent and intensity of the associational links with ARI symptoms differ depending on the composition of the physical and social context of a given child and these form a range of biological, social and environmental components of risks. Thus, in this study, we examined the effects of environmental factors of two provinces of Nigeria, including season, community orphan and vulnerable children rates (OVC), household poverty, and lifestyle factors and assessed their associations with likelihoods of ARI symptoms [14,15].

## 2. Methods

### 2.1. Study Design and Sampling Technique

We employed cross-sectional data from the 2013 Nigeria Demographic and Health Survey (NDHS), a nationally representative set of data that used a national probability sample of households and involved a three-stage cluster sampling technique. Nigeria is stratified into 36 states and the capital city Abuja; thus, there are 37 entities that form six geopolitical regions. The first stage of the three-stage cluster sampling technique was as follows: the primary sample unit (PSU) was derived from the 2006 Nigeria population census enumeration areas (EAs). Each PSU corresponded to the smallest geographic unit, namely a neighborhood in an urban area or a village in a rural area, which gave a total of 896 clusters. Each primary sample unit was then selected based on a probability, proportional to the population size approach. The second stage of the three-stage cluster sampling technique involved households being selected within the PSUs by systematic sampling within the selected clusters. The third stage involved the distribution of households within each state based on a probability proportional to the urban and rural areas, using a total of 40,680 households. Among the successfully sampled households, information on 31,482 under-five children (a combination of dead or alive children) were available, with a 97.6% response rate. In this study, we present community as a sample cluster (i.e., PSU), usually a village or urban census block. We restricted the analyses to children who were under five and alive. The final sample consisted of 28,596 pre-school children [16]. Further information about this dataset can be found in other published articles [17]. 

### 2.2. Ethical Approval 

The dataset is available online from ICF international, Rockville, MD, USA [18]. The study protocol used secondary data analysis of the DHS and was approved by the Research Ethics Committee of National Yang-Ming University in Taipei, Taiwan (YM104025E-2).

### 2.3. Operationalization of the Major Measures

#### Dependent Variable

The symptoms of acute respiratory infection (ARI) were an outcome variable that was defined and measured by the 2013 NDHS [18], a woman’s health questionnaire administered to eligible women (15–49 years). This revealed information on the respiratory health of children aged from 0 to 59 months. Mothers were asked whether their children under the age of 5 had been ill with a cough in the two weeks preceding the survey. If the child had suffered from a cough, they were also asked if the child had suffered from short rapid breathing and/or a fever during the 2 weeks prior to the survey. Children who met all three of the abovementioned criteria were regarded as having ARI and were coded with a value of 1; otherwise, they were coded with a value of 0.

### 2.4. Individual-Level Explanatory Variables

*Household characteristics:* Cooking methods were derived based from, and based on, two variables, namely the major type of cooking fuel used in the households and the place where cooking occurred. The variable was grouped into five categories. These were households using kerosene/charcoal outdoors or in a separate place, biomass cooking indoors, biomass cooking outdoors in a separate place, kerosene/charcoal cooking in the house and other less polluting cooking methods. The tobacco smoking status of household members was also classified according to whether the mother or any household member were smokers or not [17]. 

*Household poverty* was derived from the household wealth index, which consists of five quintiles of asset-based measurements for the country. This measure was categorized into either higher than 40% or 40% and lower. That is, a household was coded as being in poverty if it was within the lower two quintiles.

*Orphans and vulnerable children (OVC)* were identified as those who had experienced the death of a family member, who had been ill for at least 3 months in the past 12 months, or those from a household that included a member who has been ill for at least 3 months in the past 12 months. We used this set of variables related to vulnerability to assess if a child had been exposed to such experiences and if this was true the child was coded 1. Otherwise the child was encoded as 0. Based on the premises of earlier studies [19,20], a number of individual covariates were also included in the study. These were related to family socio-demographic background and consisted of the child’s gender (male and female), age (<1, 1–2, and 3–5 years), birth order (1–3, 4–6 and >6), their mother’s education (no education, primary education, or secondary education) and their mother’s religion (Christianity, Islam or Traditionalist). These covariates have often been linked to symptoms of ARI in earlier studies [10,17,21]. 

Table 1 shows the categorization and distribution of the regional community control variables from the 2013 DHS dataset for Nigeria. This information, derived from the Nigerian 2013 DHS dataset, consists of the categorization and distribution of the community variables. For the purpose of this study, a derived covariate, the community OVC status, was constructed by aggregating the household and individual survey data for the primary sampling unit and further dichotomized into lower and upper levels of household poverty. Furthermore, the computation of the integral regional variables was done by extracting from the population and housing data, in order to obtain each subject’s province of residence. For the purpose of this study, we focused on the North-Western and South-Southern provinces. In addition, seasonal factors were dichotomized into the wet season and the dry season, depending on the month in which each interview took place, and that province’s specific seasonal variation. 

### 2.5. Analytical Strategy

The analyses first characterized the distribution of the regional communities, the household level characteristics and the children-level characteristics of the sample population, in terms of the prevalence of ARI symptoms by region of residency. We then conducted a bivariate regression analysis between each covariate and the likelihood of ARI symptoms. Due to the hierarchical structure of the DHS data (level 1 = children and level 2 = census tract/community), we employed a two-level random intercept legit model. In the null model I, it was found there was a 15% variation in ARI symptoms across the communities (*p* < 0.01) [22]. Multilevel modeling was then used to examine the simultaneous associations between regional context and ARI symptoms, adjusting for neighborhood, household and individual backgrounds. All analyses used State 13.0 updated with the glam program for random intercept multilevel models [23]. 

## 3. Results

Table 1 shows that there was a substantial difference between provinces in terms of ARI prevalence in Nigeria, namely 5.7% in the North-Western province and 1.4% in the South-Southern province. However, the characteristics of the children in the two provinces were quite similar. Furthermore, the maternal and household characteristics differed significantly between the two provinces. For example, two-thirds (66.9%) of mothers in the North-Western province versus less than one-tenth (7.4%) in the South-Southern province had no education. Furthermore, more than two-thirds of children in the North-Western province (68.0%) versus about one-ninth in the South-Southern province (11.5%) were below the designated poverty level. Finally, a significantly higher percentage of cooking with biomass fuel indoors occurred in the North-Western province (55.4%), compared to the South-Southern province (5.3%).

Table 2 presents the odds ratios (ORs) and 95% confidence intervals (CIs) for the explanatory variables and the risk of ARI symptoms. For the crude model, the unadjusted association between each explanatory variable and the ARI symptoms outcomes are presented. For the adjusted model, a multivariate framework is shown, wherein both individual-level and provincial-level factors are included. Even after adjusting for child and maternal characteristics, young children in the North-Western province were significantly more likely to have a higher risk of ARI symptoms (aOR = 2.86, *p* < 0.001). Furthermore and specifically, the dry season (aOR = 1.42, *p* < 0.05) and household poverty (aOR = 1.42, *p* < 0.01) were also significantly associated with an increased risk of ARI symptoms. When the children’s characteristics were considered, there was an increased likelihood of ARI symptoms significantly associated with children aged 1–2 years old (aOR = 2.16, *p* < 0.01) and children from higher up the birth order (aOR = 1.58, *p* < 0.01), compared to their counterparts.

## 4. Discussion

When trying to address gaps in the literature regarding the hypothesis wherein one possible mechanism that influences children’s health is the different environmental contexts that they are embedded in, it is clear that this hypothesis has seldom been investigated. In particular it has not been addressed in terms of whether a country’s differential environmental conditions can be independently associated with a child’s health; specifically, in the case of whether a child suffers from ARI symptoms. This study employed a nationally representative survey from Nigeria, and has revealed the relevance of independent context-specific environmental conditions on children’s ARI risks, using multi-level logit models.

Guided by the exposure-disease-stress model [4], our analysis has empirically revealed that differential environmental risk factors are present, which are specific to given provinces and are significantly linked to variation in ARI risks among preschool children, with the magnitude of this effect varying within a specific country. These findings underscore the importance of environmental risk factors that are specific to the region of residence and these seem to play a pertinent role in influencing the risk of ARI symptoms, hence the resulting differential determinants. For instance, preschool children from the North-Western province compared to the South-Southern province have a significantly higher risk of ARI symptoms. In addition, the dry season also creates a higher risk of ARI symptoms among preschool children. In order to combine these two pieces of information in a way that ought to aid operationalization of environmental risk factors specific to each region, there is a need to understand the context-specific ARI risk factors in more detail. Analysis of the Nigerian DHS dataset provided evidence that there are differential individual and environmental risk factors between the North-Western province and the South-Southern province and these are associated with ARI symptoms of children in these regions. These context specific differences are possibly the result of the different political milieu and the high level of religious unrest present in the North Western region compared to its counterpart, the South Southern region [24]. In an analysis not shown here, within these provinces, we have found pre-school children are at a higher risk of ARI symptoms due to a high rate of community orphanhood. The political unrest in the North-Western region of Nigeria may have resulted in greater social and economic spatial development inequality compared to the South-Southern region of Nigeria, placing the former province at a significant disadvantage to the other [25,26]. Consistent with previous findings, other regional specific factors, such as seasonal factors, are also important; specifically, the dry season has an independent and statistically significant association with ARI symptoms [27]. This can be explained by the geographic location of the Northern regions of Nigeria, which results in an exposure to higher levels of dust exposure, including sand storms from the Gulf of Guinea area [12]. 

A prior study [22] suggested a positive association between household cooking methods and risks of ARI symptoms amongst children, when indoor-biomass is used by households; this is quite consistent with the findings in the present study. The associations between biomass fuel smoke exposure and ARI symptoms is considered causal [28,29]. Kerosene and charcoal are fuels with a medium level of pollution and Kilabuko and Nakai suggested that it is possible that emissions from these fuels are of such a level that changing to them from a high pollutant fuel such as biomass fuel will provide substantial health hazards [19]. 

Moreover, our analysis is consistent with prior findings that the prevalence of ARI symptoms is particularly serious among younger children who are aged 1–2 years old [14]. However, it can be further argued that this age group will have developed a higher sense of bonding with, and recognition of, their mothers and thus will be more demanding to be backed (carried on the mother’s back) or cuddled by their mothers whilst cooking. 

This will increase their level of exposure to smoke, which will lead to a higher risk of ARI. In addition, overcrowding is another major factor that affects ARI symptoms in children. Specifically, our results show that children who have a higher birth order, and thus have more brothers and sisters, are at higher risk of having ARI symptoms [17]. Finally, OVC status also results in a higher risk of ARI symptoms but this is not statistically significant in this study [10]. Household poverty, defined as the family having a household wealth less than 40% of the national level of household wealth, is a significant risk factor for ARI symptoms among children. This supports the common hypothesis that ARI risk is likely to be reduced as the contextual economy and spatial development improves [17]. According to Nigerian literature, at the advent of socioeconomic development, the North-Western and South-Southern regions were specifically characterized as having higher morbidities and mortalities among preschool children. Furthermore, these regions were not used as the benchmark for free education, agricultural settlement and industrial development unlike the Western regions of Nigeria [30]. Consequently, parents in these areas are relatively less educated and are less likely to know what conditions create or exacerbate ARI symptoms. However, in our analysis, even though there are a significantly higher percentage of educated mothers in the South-Southern region, there were no significant associations between maternal education and ARI symptoms. This was also true for religion and ARI symptoms. This may be a result of the social and economic spatial development inequality between the North-Western and South-Southern Nigerian provinces, which has led to one province having more children with ARI symptoms than the other [25,26] .

Regardless of the above, our findings need to be interpreted in consideration of the limitations of this study. Firstly, due to the cross-sectional nature of the data, we were unable to infer causal relationships. Secondly, an underestimation of the prevalence of ARI may have occurred due to a recall bias. Underreporting of ARI symptoms raises another possible concern among mothers who lived in households using biomass fuel; this is because the majority of such mothers are more likely to lack an awareness of ARI symptoms and fail to identify them during the 2 week recall period, as the majority of mothers with low socioeconomic status use biomass fuel cooking. Finally, it is always important to realize that endogeneity challenges self-reported measures. Nonetheless, our study does provide, using a multilevel analytical perspective, important insights into the various identified environmental risks that are relevant to these particular provinces in Nigeria, a sub-Saharan African country with high levels of ARI compared to other countries in this region.

## 5. Conclusions

Given the limitations of this study, the findings from this study address several important gaps in literature. The population based sample size of the Nigerian DHS survey allowed extensive analyses of subgroups and their differential effects of environmental exposures on children’s health outcomes; additionally, the strategic design and sampling procedures made essential avenues available for assessing the associations between households/individual characteristics region-specific influences and ARI symptoms.

Most existing literature addressing ARI symptoms among pre-school children in SSA countries is descriptive and at the individual level, only a handful have explored certain neighborhood-level influences. This study extended these efforts by examining provincial specific mechanisms through which differences in household lifestyles and individual characteristics in specific provincial environments were associated with differences in ARI symptoms.

Moving forward, in broader detail than possible prior research, the wealth of information generated from the DHS about several key concepts, such as seasonal factors, OVC status, and neighborhood influences, the relevant information has provided room for examinations of characteristics related to ARI symptoms among preschool children.

The findings on the provincial-specific variables and their relationship to ARI symptoms within the context of this study have been able to advance our knowledge. This will inform policy makers to take a context-specific approach in regard to the cause of reduction of ARI prevalence. To achieve minimum levels of health for economic development to take root across these provinces, future health policies that aim to promote child health should be taken into consideration, with tailored interventions regarding ARI control among preschool children.

## Figures and Tables

**Table 1:** Relationship between children and household variables and the likelihood of symptoms of acute respiratory infection (ARI) among Nigerian preschool children from the North-Western and South-Southern communities, 2013 NDHS.

Variables	North-Western		ARI	South-Southern		ARI
	(N = 5856)	(%)	Prevalence	(N = 3498)	(%)	Prevalence
**Seasonal characteristic**						
Wet season	4052	69.19	4.26	2114	60.43	1.83
Dry season	1204	30.81	7.00	1384	39.57	1.61
**Household characteristics**						
Cooking method						
In-house kerosene or charcoal	98	1.67	1.94	750	21.46	1.11
In-house biomass fuel	3243	55.41	5.38	184	5.26	2.58
Outdoor or separate use of biomass fuel	2309	39.45	4.76	1959	56.05	1.58
Outdoor use kerosene/charcoal/others	175	2.99	5.59	473	13.53	2.20
Others	28	0.48	0.00	129	3.69	3.47
Household poverty						
Yes (40% or lower)	1873	31.98	4.46	3096	88.51	1.62
No (>40%)	3983	68.02	5.37	402	11.49	2.55
**Maternal characteristics**						
Maternal education						
No education	3918	66.91	4.99	260	7.43	0.87
Primary education	962	16.43	6.00	1119	31.99	1.36
Secondary and above	976	16.67	4.59	2119	60.58	1.98
Mother’s religion						
Christian	1017	17.43	5.12	3383	97.13	1.74
Islam	4770	81.75	5.05	78	2.27	0.00
Traditionalist	48	0.82	6.71	21	0.60	0.00
**Child’s characteristics**						
OVC status						
No	5068	95.77	4.95	3236	93.80	1.68
Yes	248	4.23	7.73	217	10.00	2.36
Gender						
Male	3038	51.88	4.72	1746	49.91	1.63
Female	2818	48.12	5.44	1752	50.09	1.82
Age (years)						
<1	1260	21.52	5.93	762	21.78	1.61
1–2	1184	20.22	7.61	735	21.01	2.97
3–5	3412	58.27	3.81	2001	57.20	1.30
Birth order						
1–3	2695	46.02	4.12	1974	56.43	1.72
4–6	1916	32.72	5.18	1094	31.28	1.69
>6	1245	21.26	5.07	430	12.29	1.91
**Neighborhood characteristics**						
Low 25% communty status of orphan and vulnerable children (OVC) rate	3108	53.07	4.13	1775	50.74	1.67
High 25% communty OVC rate	2748	46.93	6.12	1723	49.26	1.78
**Outcome measure**						
Symptoms of ARI			5.67			1.43

*Note*: Unweighted N’s and weighted percentages and means are reported. Percentages may not add up to 100 due to rounding off. ARI prevalence was calculated by dividing the number of ARI cases in a specific category by the number of children measured in this specific category.

**Table 2:** Results from the multilevel logistic regressions for the odds of ARI symptoms among Nigerian preschool children from the North-Western and South-Southern communities, 2013 Nigeria Demographic and Health Survey (NDHS).

Explanatory Variables	Crude Model	Adjusted Model
	uOR (95% CI)	aOR (95% CI)
**Regional characteristic**		
Region of residence (ref = South-Southern)		
North-Western	2.94 (2.04–4.24) ***	2.86 (1.62–5.04) ***
**Seasonal characteristics**		
Seasonal factor (ref = wet season)		
Dry season	1.46 (1.06–2.03) *	1.42 (1.02–1.97) *
**Household characteristics**		
Cooking method (ref=in house kerosene/charcoal)		
In-house biomass fuel	4.85 (2.38–9.89) ***	1.76 (0.84–3.92)
Outdoor or separate use of biomass fuel	3.02 (1.49–6.14) **	1.55 (0.75–3.40)
Outdoor use kerosene/charcoal/others	2.60 (1.14–5.91) *	2.25 (1.00–5.05) *
Others	1.84 (0.47–7.20)	2.86 (0.87–8.25)
Household poverty (ref = no)		
Yes	1.67(1.29–2.18) ***	1.42 (1.01–1.99) *
**Maternal characteristics**		
Maternal education (ref = no education)		
Primary education	1.36 (0.93–2.00)	1.07 (0.75–1.54)
Secondary and above	0.76 (0.56–1.03)	1.22 (0.81–1.83)
Mother’s religion (ref = Christian)		
Islam	2.16 (1.61–2.90) ***	0.91 (0.56–1.45)
Traditionalist	2.85 (0.97–8.34) *	1.23 (0.28–5.35)
**Child’s characteristics**		
OVC status (ref = no)		
Yes	1.36 (0.88–2.09)	1.41 (0.88–2.26)
Gender (ref = male)		
Female	1.06 (0.85–1.30)	1.12 (0.88–1.42)
Age (ref = 3–5 years)		
<1	1.49 (1.14–1.94) **	1.50 (1.11–2.02) **
1–2	2.04 (1.59–2.61) ***	2.16 (1.64–2.84) ***
Birth order (ref = 1–3)		
4–6	1.21 (0.95–1.54)	1.16 (0.88–1.54)
>6	1.41 (1.07–1.86) *	1.58 (1.15–2.15) **
**Neighborhood Characteristics**		
Community OVC rate (ref = low 25%)		
High 25% community OVC rate	1.36 (0.93–2.00)	1.30 (0.95–1.78)
Model statistics		Coeff (SE)
Log likelihood		−1201.7363
**Random variance**		
Intra-class correlation (ICC)		0.08 *
Variance between neighborhoods		0.56 *** (0.09)

*Note*: Intra-class correlation (ICC) measures the degrees of clustering with random intercepts. The correlation of the two-level multilevel logistic regressions was calculated by σ_μ_^2^/(σ_μ_^2^ + π^2^/3), where σ_μ_^2^ denotes neighborhood-level variance. uOR refers to unadjusted odds ratios; aOR refers to adjusted odds ratios for covariates and CI refers to confidence interval; * *p* < 0.05; ** *p* < 0.01; *** *p* < 0.001.
